# Role of preoperative zinc, magnesium and budesonide gargles in Postoperative Sore Throat (POST) - a randomised control trial

**DOI:** 10.1186/s12871-024-02534-5

**Published:** 2024-05-01

**Authors:** Aakanksha Bhanwra, Sanjeev Palta, Richa Saroa, Puja Saxena, Sangeeta Bhanwra, Aditi Jain

**Affiliations:** 1 Department of Anaesthesia and Intensive Care, Government Medical College and Hospital, Sector 32, Chandigarh, 160030 India; 2 Department of Anaesthesia and Intensive Care, Dr. B.R. Ambedkar State Institute of Medical Sciences, Sector 57, Sahibzada Ajit Singh Nagar, Punjab, 160055 India; 3 Department of Pharmacology, Government Medical College and Hospital, Sector 32, Chandigarh, 160030 India

**Keywords:** Post-operative, Sore throat, General Anaesthesia, Endotracheal intubation, Magnesium, Zinc, Budesonide

## Abstract

**Background:**

Post-operative sore throat (POST) has an incidence ranging from 21 to 80%. To prevent the development of POST, several pharmacological measures have been tried. Aim of this study was to compare the efficacy of preoperative zinc, magnesium and budesonide gargles in reducing the incidence and severity of POST in patients who underwent endotracheal intubation for elective surgeries.

**Methods:**

We conducted a prospective, randomized, double-blind, controlled equivalence trial in 180 patients admitted for elective surgical procedures under general anaesthesia. Patients were randomised into three groups; group Z received 40 mg Zinc, group M received 250 mg Magnesium Sulphate and group B received 200 µg Budesonide in the form of 30 ml tasteless and colourless gargle solutions. Sore throat assessment and haemodynamic recording was done postoperatively at immediate recovery (0 h) and 2, 4, 6, 8, 12 and 24 h post-operatively. POST was graded on a four-point scale (0–3).

**Results:**

POST score was comparable at all recorded time points i.e. 0,2,4,6,8,12 and 24 h. Maximum incidence was seen at 8 h in group B (33.3%) and the minimum incidence was at 24 h in group Z (10%) (*p* > 0.05). It was found that the incidence of POST was more in the surgeries lasting longer than 2 h in all groups. This difference was found to be statistically significant in Groups M and B. The incidence of POST was found to be comparable between laparoscopic and open procedures.

**Conclusion:**

Magnesium, zinc and budesonide have an equivocal effect in the prevention of POST at different time points. The incidence of sore throat increases significantly in surgeries lasting more than two hours if magnesium or budesonide have been used as premedicant. Duration of surgery is an independent predictor for POST.

**Trial registration:**

CTRI/2021/05/033741 Date-24/05/2021(Clinical Trial Registry of India).

## Background

Emergence from general anaesthesia may be accompanied by numerous physiological disturbances involving multiple organ systems. Most commonly encountered post-operative problems after general anaesthesia include - pain, nausea and vomiting, thirst, drowsiness, hoarseness, sore throat, cold, confusion and shivering [1]. Amongst these, post-operative sore throat (POST) has an incidence ranging from 21–80%. It has been seen to last for even 12–24 h after surgery, leading to prolonged hospital stay, especially in day care ﻿surgeries [2]. Cough and hoarseness of voice may be concomitant with POST leading to increased restlessness, anxiety and discomfort in patients [3]. 

While there is no existing standard of care for the prevention of POST, various strategies have been used to prevent it’s development with varying degree of success. Nonpharmacological measures include smaller sized tracheal tubes, careful airway instrumentation, minimizing the number of laryngoscopy attempts, adequate relaxation prior to intubation, gentle oropharyngeal suctioning, filling the cuff with an anaesthetic gas mixture or normal saline, minimizing intracuff pressures and use of video laryngoscopy.^4,5^.

Amongst the pharmacological measures, steroids, azulene sulfonate, aspirin, ketamine, magnesium sulphate, liquorice, benzydamine hydrochloride, zinc and lignocaine have been studied [6]. The route of administration of drugs has been multi-variate viz. intravenous, inhalational and topical routes that primarily include gargles, local sprays and lozenges.^7,8^ Local routes of administration allow immediate release of the drug which adheres to the pharyngeal wall and decreases oedema. Literature pertaining to the comparative effects of zinc, magnesium and budesonide on the prevention of POST is sparse. Hence, this study was designed to compare the efficacy of preoperative zinc, magnesium and budesonide gargles in reducing the incidence and severity of POST in patients who underwent endotracheal intubation for elective surgeries. The objective of the study was to assess the effect of preoperative zinc, magnesium or budesonide on the severity of (POST) and to study the adverse effects associated with administration of the study drugs.

## Methods

This prospective, double blind, three arm intervention, controlled equivalence trial was carried out in a tertiary care hospital in North India (Government Medical College and Hospital, Chandigarh). After receiving approval from institutional ethics board (GMCH/IEC/2020/438/26) it was registered in the national registry (CTRI/2021/05/033741). 180 patients admitted for elective surgical procedures under general anaesthesia with endotracheal intubation (GETA) were screened and recruited between May 2021 and June 2022 as per the inclusion and exclusion criteria one day prior to surgery during preanesthestic evaluation in their respective surgical wards. Patients were explained the grading for POST and were told that they would be asked about the same at immediate recovery (0 h), 2, 4, 6, 8, 12 and 24 h post-operatively. Patients from 18 to 60 years of age of all gender who were ASA 1 and 2 undergoing surgery of expected duration less than 6 h in supine position were recruited. Patients undergoing head and neck surgeries, having a history of pre-operative sore throat, asthma, chronic obstructive pulmonary disease (COPD), gastro-esophageal reflux disease (GERD) or cervical spine injury, having an anticipated difficult airway, parturients and morbidly obese patients were excluded. Post recruitment randomisation was done using computer generated random number table with coded, sealed, opaque envelopes to one of the following groups to receive preoperative gargling with the respective drug:

Group Z (*n* = 60) - Patients received 40 mg Zinc.

Group M (*n* = 60) - Patients received 250 mg Magnesium Sulphate.

Group B (*n* = 60) - Patients received 200 µg Budesonide.

A 30 ml solution prepared in a 50 cc syringe was used for gargling by the patients in each group. 2 ml of 20 mg/ml zinc sulphate oral solution was diluted with plain water upto 30 ml for the patients in the zinc group making the final dose delivered as 40 mg. For the magnesium group, 0.5 ml of magnesium sulphate ampoule (500 mg/ml) was diluted in plain water upto 30 ml for a final dose of 250 mg. The patients in budesonide group received 1 ml of budesonide suspension taken upto 30 ml in plain water to administer 200 µg budesonide. All the three solutions were tasteless and identical in appearance (colourless solutions).

On the day of surgery, after recruitment, the enrolled patients were randomized into three groups with the help of computer-generated random number tables using opaque sealed envelopes prepared by an anaesthesiologist, not part of the study. This person prepared unlabelled, identical-looking 30 ml drug in a 50 cc syringe for all patients. The person involved in administering the drugs did not assess the patients for POST after the surgery. Patients were also unaware of the study drug (blinded) as all three preparations were tasteless and identical in appearance. Thus, both the patient and the investigator were blinded to the study drugs. The patients were made to gargle the study drugs in sitting position with neck extension for 10 min in the preoperative room, 20 min before induction of general anaesthesia. They were made to spit out the solution in a pan completely after gargling. The preoperative hemodynamic parameters and sore throat assessment was done prior to administration of the study drugs and was recorded in the prescribed proforma. The patients were transferred to the operating room and induction of general anaesthesia was done minimum ten minutes after completion of gargling. A repeat recording of the hemodynamic parameters was taken before the induction. All the patients were attached to ASA standard monitors for continual monitoring.

A standardized general anaesthetic technique was followed in all patients. All patients were preoxygenated and induction was achieved 10 min after completion of drug administration in the patients with intravenous (IV) morphine 100 µg/kg and propofol 2 mg/kg. Endotracheal intubation was facilitated with IV non depolarizing muscle relaxant vecuronium bromide 0.1 mg/kg and IV lignocaine 1 mg/kg and the trachea was intubated with a (soft seal) cuffed sterile polyvinyl chloride tracheal tube of appropriate size using a Macintosh laryngoscope. Endotracheal intubation was performed only by experienced anaesthesiologists (> 100 intubations) after adequate muscle relaxation. All the patients were reversed at the end of the surgery with IV neostigmine 50 µg/kg and glycopyrrolate 10 µg/kg and trachea was extubated if the patient was deemed fit for extubation. All the patients were transferred to post-operative care unit (PACU) for observation and postoperative management. Post-operative pain was managed by the acute pain services (APS) team as per institutional protocol. In PACU, sore throat assessment and haemodynamic recordings were done at immediate recovery (0 h), 2, 4, 6, 8, 12 and 24 h post-operatively. POST was graded on a four-point scale (0–3): 0 = no sore throat; 1 = mild sore throat (complains of sore throat only on asking); 2 = moderate sore throat (complains of sore throat on his/her own); and 3 = severe sore throat (change of voice or hoarseness, associated with throat pain). Other adverse effects, if any, were noted. All the observations were recorded in prescribed proforma and were subjected to appropriate statistical analysis.

### Statistical analysis

The sample size was calculated by a reference trial of Farhang et al. in which zinc was compared against a placebo [9]. Taking the expected per cent POST incidence in all the drug groups as 7% each and the equivalence criteria to be an absolute value of 20%, the sample size was calculated as 51 in each arm with 95% power and a two-sided confidence interval of 95%. To compensate for any dropouts and to make a round figure, we decided to enrol 190 patients.

The normality of the quantitative variables was tested with the Shapiro-Wilk test/ Kolmogorov Smirnov tests of normality. Continuous data, if normally distributed was written in the form of its mean and standard deviation and when skewed was presented in the form of its median and interquartile range. Group comparisons of values of skewed data were made with Kruskall-Wallis test followed by the Mann-Whitney test for 2 groups. ANOVA followed by a post hoc multiple comparisons test was carried out for comparison of normally distributed data. The Spearman correlation coefficient was calculated to see the relationship of different variables with duration. Multinominal logistic regression analysis was carried out to find independent predictors of post-operative sore throat. Categorical variables were reported as counts and percentages. Group comparisons were made with the Chi-Square test or Fisher’s exact test. Sub group comparison of type of surgery was carried out for POST. Duration of surgery was divided into two categories (less than/ equal to 2 h and more than 2 h) according to median of duration. A P-value < 0.05 was considered significant. All the statistical tests were two-sided and were performed at a significance level of α = 0.05. Analysis was conducted using IBM SPSS Statistics (version 25.0).

## Results

**Fig. 1:**
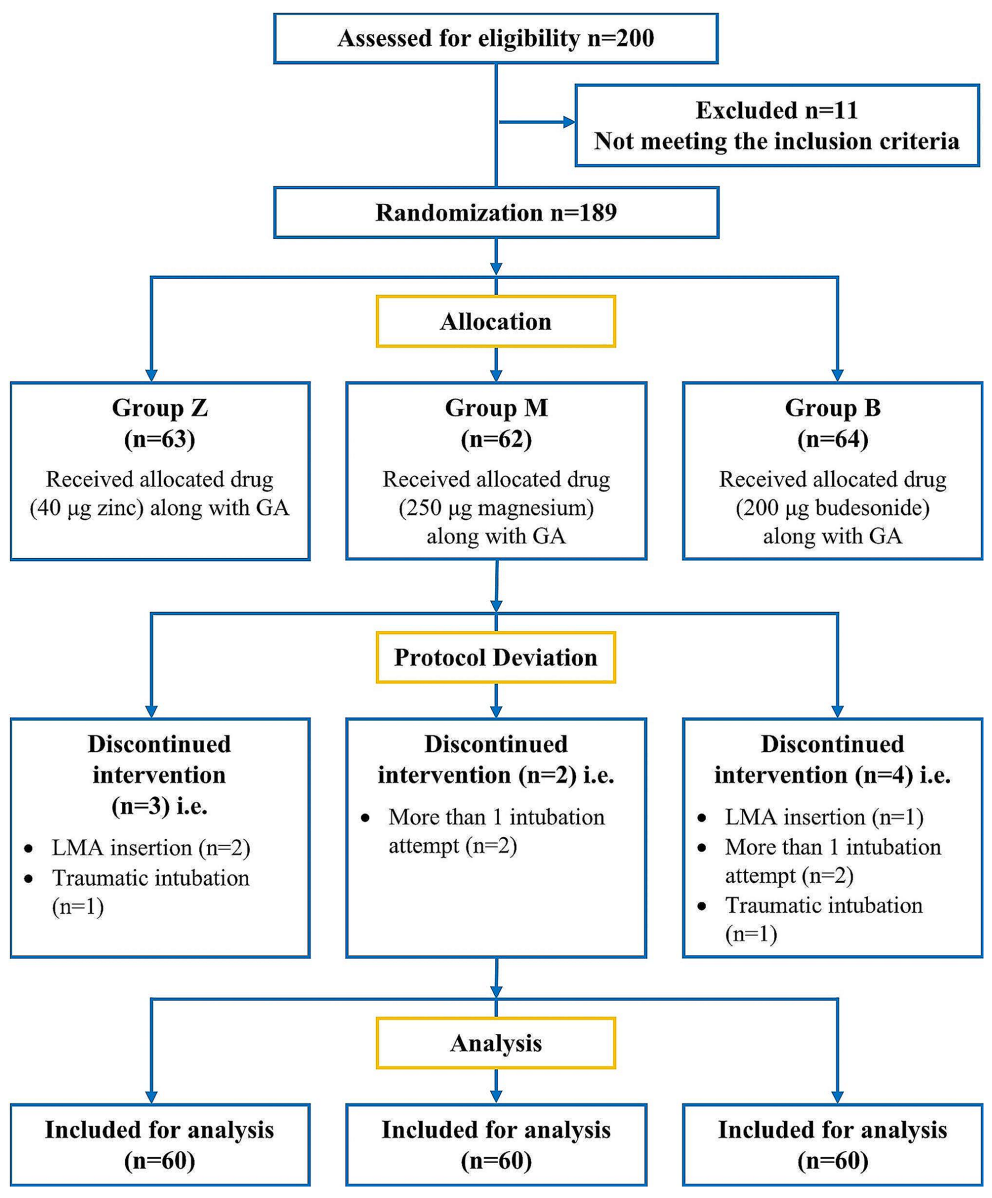
Consort diagram

Two hundred patients planned for elective surgery under general anaesthesia with endotracheal intubation were screened. (Fig. 1) Eleven were excluded as per the exclusion criterion and 189 patients were randomised to the three groups. There was protocol deviation in 3 patients in Group Z, 2 patients in Group M and 4 patients in Group B. Finally, 60 patients in each group were included for analysis.

**Fig. 2:**
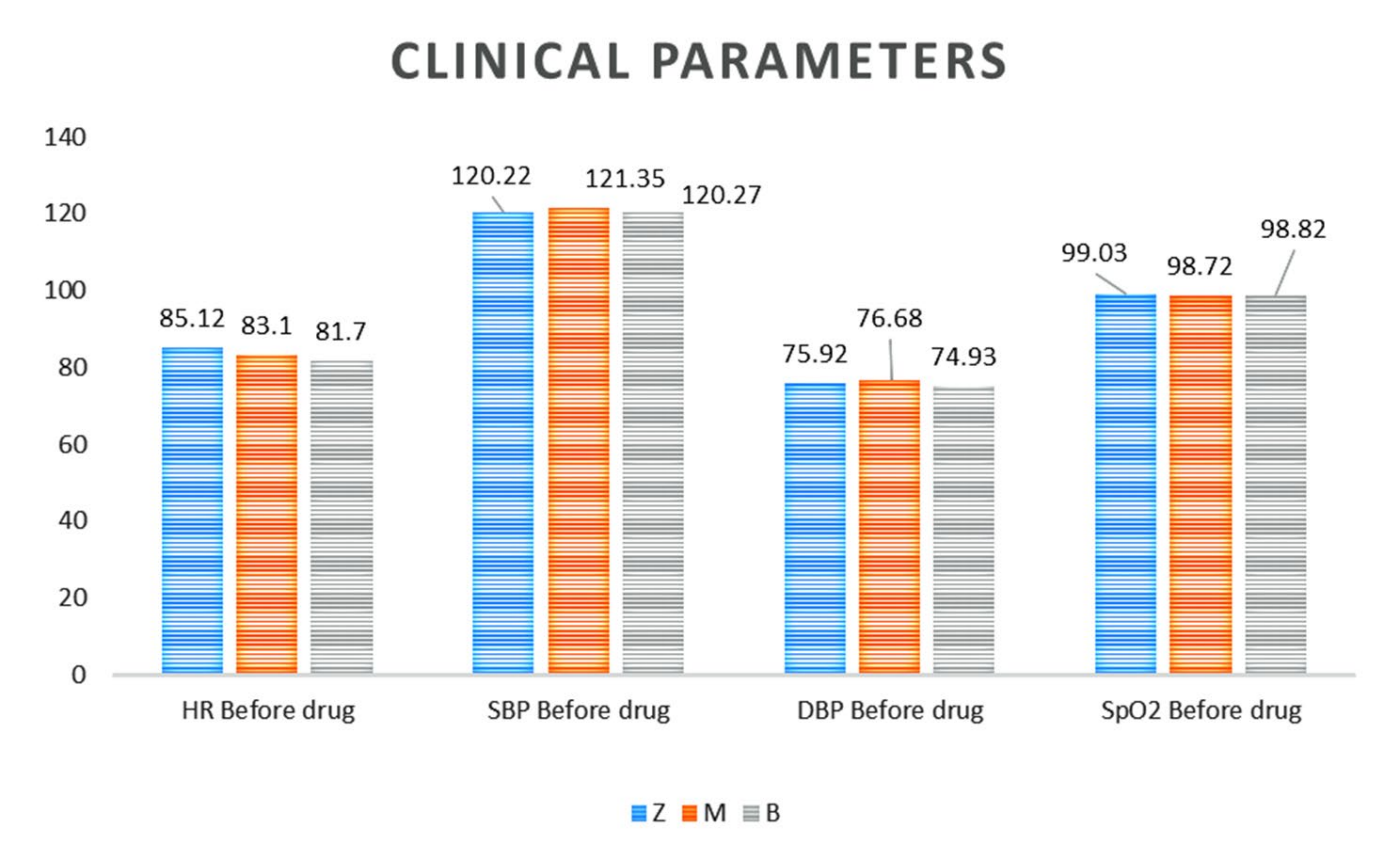
Vital parameters recorded prior to drug administration

The patients in all three groups had a similar demographic profile. (Table 1) The vital parameters recorded prior to drug administration were comparable. (Fig. 2)

**Fig. 3:**
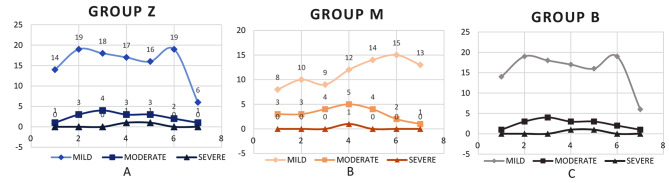
Number of patients with mild, moderate and severe POST over time

In PACU, we graded POST on a four-point scale (0–3): 0 = no sore throat; 1 = mild sore throat (complains of sore throat only on asking); 2 = moderate sore throat (complains of sore throat on his/her own); and 3 = severe sore throat (change of voice or hoarseness, associated with throat pain). Amongst the patients who complained of a sore throat, it was seen that the score was comparable at all recorded time points i.e. 0,2,4,6,8,12 and 24 h. (Table 2). Maximum incidence of sore throat was seen at 8 h in group B (33.3%) and the minimum incidence of sore throat was seen at 24 h in group Z (10%) although not found to be statistically significant. (Fig. 3)

When the incidence of sore throat was investigated in surgeries lasting less than versus more than 2 h it was found that the incidence of POST was more in the surgeries lasting longer than 2 h in all groups. Duration of surgery was also found to be an independent predictor for development of sore throat with an OR of 3.4. This difference was found to be more than double and statistically significant in Groups M and B. (Table 3) There was a difference of more than 10% in the incidence of patients having sore throat when premedicated with magnesium or budesonide as compared to those who were premedicated with zinc. The risk reduction of Group Z was 10.86% when compared to group B and 12.86% when compared to group M.

When comparing the incidence of POST between laparoscopic and open procedures the incidence of POST was found to be comparable.

There were no significant perioperative hemodynamic events. The vitals were maintained within 20% of baseline throughout the intraoperative period. No adverse effects were reported in either of the study groups.

**Table 1:** Comparison of patient characteristics in the three groups. The data is represented as mean ± standard deviation or number (N = number of patients)

Characteristics	Group Z	Group M	Group B	P-Value
	*N* = 60	*N* = 60	*N* = 60	
Age (years)	37.5 ± 11.07	40.4 ± 10.40	38.58 ± 12.16	0.362
Female	38	45	51	0.025
Male	22	15	9	
ASA I	43	42	41	0.712
ASA II	17	18	19	
Duration (hours)	2.14 ± 0.90	2.11 ± 0.73	2.27 ± 0.77	0.454

**Table 2:** Severity of post in the three groups at different evaluation times

TIME	GROUP Z (*N* = 60)			GROUP M (*N* = 60)			GROUP B (*N* = 60)			P-VALUES
	MILD (GRADE 1)	MODERATE (GRADE2)	SEVERE (GRADE 3)	MILD (GRADE 1)	MODERATE (GRADE2)	SEVERE (GRADE 3)	MILD (GRADE 1)	MODERATE (GRADE2)	SEVERE (GRADE 3)	
0 h	14(23.3%)	1(1.7%)	0(0%)	8(13.3%)	3(5%)	0(%)	11(18.3%)	3(5%)	0(0%)	0.564
2 h	19(31.7%)	3(5%)	0(0%)	10(16.7%)	3(5%)	0(0%)	16(26.7%)	3(5%)	0(0%)	0.434
4 h	18(30%)	4(6.7%)	0(0%)	9(15%)	4(6.7%)	0(0%)	17(28.3%)	1(1.7%)	0(0%)	0.179
6 h	17(28.3%)	3(5%)	1(1.7%)	12(20%)	5(8.3%)	0(0%)	19(31.7%)	2(3.3%)	0(0%)	0.507
8 h	16(26.7%)	3(5%)	1(1.7%)	14(23.3%)	4(6.7%)	0(0%)	20(33%)	2(3.3%)	1(1.7%)	0.795
12 h	19(31.7%)	2(3.3%)	0(0%)	15(25%)	2(3.3%)	0(0%)	16(26.7%)	0(0%)	1(1.7%)	0.573
24 h	6(10%)	1(1.7%)	0(0%)	13(21.7%)	1(1.7%)	0(0%)	10(16.7%)	1(1.7%)	0(0%)	0.549

**Table 3:** Incidence of POST in relation to duration of surgery in the three groups. The data is represented as number of affected patients (incidence in %)

INCIDENCE OF POST IN RELATION TO DURATION OF SURGERY			
GROUP	DURATION OF SURGERY		P-value
	<=2 h	> 2 h	
**Z**	16 (43%)	12 (57%)	0.316
	*N* = 39	*N* = 21	
**M**	12 (30%)	14 (70%)	0.0032
	*N* = 40	*N* = 20	
**B**	11 (31.4%)	17 (68%)	0.0051
	*N* = 35	*N* = 25	

(N = number of patients)

## Discussion

Post-operative sore throat (POST) after general anaesthesia is a cause of significant concern for all anaesthesiologists in the postoperative period due to its high incidence.^3,10,11^ We aimed to compare the effects of preoperative zinc, magnesium or budesonide gargles on the incidence of POST in patients scheduled to undergo elective surgery under general anaesthesia. The objectives included the assessment of the effect of preoperative zinc, magnesium or budesonide gargles on the severity of POST and the study of the adverse effects associated with the administration of these drugs. Our hypothesis was that zinc, magnesium and budesonide would have an equivocal effect in reducing the incidence of POST.

In the present study, preoperative zinc, magnesium and budesonide gargles had an equivocal effect in reducing the incidence of POST, as the incidence of sore throat in the three groups was found to be comparable at all time points (0, 2, 4, 6, 8, 12 and 24 h).

Zinc and budesonide have emerged as newer agents for the prophylaxis of POST. Zinc is a micronutrient and immune system modulator, with anti-oxidant and anti-inflammatory properties.^12,13^ Budesonide is a potent glucocorticoid and weak mineralocorticoid and has an imminent role in reducing the number of inflammatory cells and mediators in the airways thus decreasing airway hyperactivity.^3,14^ On the other hand, magnesium is a time-tested agent that has been given through various routes due to its analgesic and anti-inflammatory effects, especially in the presence of alkaline pH. Magnesium is highly concentrated in inflamed tissues and has minimal systemic absorption leading to a prolonged action and less side effects.^15,16,17^.

Two previous studies have been done for assessing the effect of zinc lozenges. Farhang et al. conducted a prospective randomized, double-blinded, placebo-controlled study in seventy-nine patients undergoing low- or moderate-risk surgery with endotracheal intubation in 2018. The authors reported that administration of a single dose of 40-mg zinc lozenge 30 min preoperatively is effective in reducing the incidence and severity of mild and moderate POST in the first 4 h after surgery [9]. Similarly, in a placebo-controlled trial by Sarkar and Mandal in eighty-eight patients undergoing surgery in 2020, the use of a prophylactic single dose 40 mg zinc dispersible tablet given 30 min before surgery correlated with a decrease in the incidence and severity of POST in the first 4 h after extubation. Severity of POST was graded from 0 to 3 and the evaluation was done at 0, 30 min, 2, 4, and 24 h. The incidence of sore throat found in their studies at less than 24 h is lower than ours but is same at 24 h. This difference might possibly due to the difference in the method of administration. At 24 h the incidence of POST in our study is similar to theirs [18]. 

Seventy patients were enrolled for a randomized, prospective, placebo-controlled trial by Borazan et al. to analyse the role of oral magnesium lozenge in reducing post-operative sore throat in 2012. The study showed that administration of oral magnesium lozenge containing 100 mg magnesium ion, 30 min before an operation, significantly reduced the incidence and severity of POST as compared to the control group, especially at 2 h and 4 h postoperatively, after general anaesthesia with laryngoscopy and orotracheal intubation. No side effects were reported [19]. Our results are in concurrence with this study with similar incidences reported at 4 h.

In a prospective randomised study in 2018, forty-six patients undergoing laparoscopic surgeries lasting < 2 h were randomly allotted into two equal groups by Tosh et al. Group A received 200 µg budesonide inhalation suspension, using a metered dose inhaler, 10 min before intubation, and repeated 6 h after extubation while Group B received no such intervention. The authors reported that inhaled budesonide was effective in causing a significant reduction in the incidence and severity of POST seen in patients following endotracheal intubation. These results are concurrent with our findings [3]. 

In a systematic review conducted by El-Boghdadly et al. in 2016, it was seen that increased duration of surgery correlated with a greater risk of postoperative sore throat [11]. In addition, 312 elective gynaecological and general surgical patients were interviewed 24 h postoperatively to determine the presence of sore throat in a prospective observational study and it was reported that increased duration of surgery contributed to increased incidence of POST [20]. The increased incidence with increasing duration of surgery is in concurrence with the findings of our study. Amongst the three groups, the incidence of POST in patients whose surgeries lasted more than two hours was significantly higher in group M and B but not in group Z. This difference between the three groups was not found to be statistically different. The authors believe it might be worthwhile investigating this difference in future studies.

Our study had a few limitations. We did not measure the cuff pressure of the tube but followed the standard institutional cuff inflation practice to keep the trial design more pragmatic. We also did not measure the serum levels of the drugs.

## Conclusions

Magnesium, zinc and budesonide have an equivocal effect in the prevention of POST at different time points. The incidence of sore throat increases significantly in surgeries lasting more than two hours if magnesium or budesonide have been used as premedicant. Duration of surgery is an independent predictor for POST.

## Future prospects

Since preoperative zinc, magnesium and budesonide gargles have an equivocal effect in reducing the incidence of POST, the choice of drug for the prevention of POST can be based on patient profile, availability of drugs and cost factor, the cheapest being magnesium. For surgeries lasting more than two hours, zinc may be investigated for its role as the agent of choice for prolonged effect wherever feasible.

The future is open for similar research to be conducted in more diverse populations and situations such as head and neck surgeries, patients with difficult airway, surgeries in prone position, parturients and in morbidly obese patient groups.

## Data Availability

The data used to support the findings of this study are available from the corresponding author upon reasonable request.

## Abbreviations

POST: Post-Operative Sore Throat
GETA: General Anaesthesia with Endotracheal Intubation
COPD: Chronic Obstructive Pulmonary Disease
GERD: Gastro-Esophageal Reflux Disease
ASA: American Society of Anaesthesiologists
IV: Intravenous
PACU: Post Anaesthesia Care Unit
APS: Acute Pain Services
ANOVA: Analysis of Variance
IBM: International Business Machines
SPSS: Statistical Package for the Social Sciences
